# Delayed Subaponeurotic Fluid Collection in Infancy: A Case Report and Literature Review

**DOI:** 10.7759/cureus.91653

**Published:** 2025-09-05

**Authors:** Katia Mauricio, Rita Alvelos, Tomás Ferrão, Margarida Martins

**Affiliations:** 1 Department of Pediatrics, Unidade Local de Saúde da Região de Aveiro, Aveiro, PRT

**Keywords:** conservative management, delayed subaponeurotic fluid collection, instrumental delivery, non-accidental head injury, scalp swelling, subgaleal haemorrhage, term infants

## Abstract

Delayed subaponeurotic fluid collection (DSFC) is a rare, benign cause of scalp swelling in infants, typically arising weeks after birth and often linked to instrumental deliveries. We report a case of a healthy infant who presented at 10 weeks with asymptomatic, unilateral parieto-occipital swelling that resolved spontaneously within four weeks. Notably, the infant experienced a bilateral biparietal recurrence at six months, a less commonly reported manifestation. Imaging confirmed subaponeurotic fluid without underlying abnormalities. This case highlights the self-limiting nature of DSFC, even when recurrent, and underscores the importance of distinguishing it from serious conditions such as subgaleal haemorrhage, cephalohematoma, or non-accidental injury. MRI was instrumental in ruling out persistent cerebrospinal fluid leakage. Awareness of DSFC’s variable presentation can prevent unnecessary interventions and parental distress. Clinicians should adopt a conservative approach while ensuring appropriate imaging in atypical or persistent cases.

## Introduction

Delayed subaponeurotic fluid collection (DSFC), also known as spontaneous subaponeurotic fluid collection (SSFC) or delayed subgaleal fluid collection, is a recently described condition that causes scalp swelling in young infants [1]. This condition is characterised by the accumulation of fluid between the galea aponeurosis and the periosteum [2-4]. It typically manifests weeks to months after birth, often presenting in healthy, well-appearing infants [5,6].

The exact cause of DSFC remains unknown. However, it is frequently associated with traumatic or instrumental deliveries, such as vacuum extraction or forceps use, prolonged or difficult labour [2], and occasionally foetal scalp electrode monitoring [7,8]. Some theories suggest DSFC may result from cerebrospinal fluid (CSF) leakage through skull microfractures, or disruption of venous or lymphatic drainage due to birth trauma [9,10]. Although its onset can be acute, DSFC is a benign condition that typically resolves spontaneously with conservative management, usually within weeks to months [11].

Given its rare yet benign nature, increased awareness of DSFC among healthcare professionals, including paediatricians and neurosurgeons, is crucial. Early and accurate recognition of DSFC can help prevent unnecessary investigations, interventions, and parental anxiety, especially by differentiating it from more serious conditions such as non-accidental injuries or life-threatening haemorrhages [6,10,12]. Importantly, while most cases resolve without recurrence, unusual presentations, such as recurrence in a different anatomical location, highlight the need to share such variations to broaden clinical understanding.

## Case presentation

A female infant, 10 weeks of age, presented to our emergency department (ED) with a two-week history of asymptomatic scalp swelling. Her mother reported that she had been otherwise well with no abnormal movements of limbs, no vomiting, and no symptoms suggestive of infection. She was feeding and sleeping normally. No history of trauma could be elicited. There was no significant family history, including bleeding diatheses. She was born vaginally and at term but required vacuum instrumentation. No history of foetal scalp electrode usage was documented. There was no bruising, scalp swelling, or cephalhematoma noted in the immediate newborn period. 

**Figure 1:**
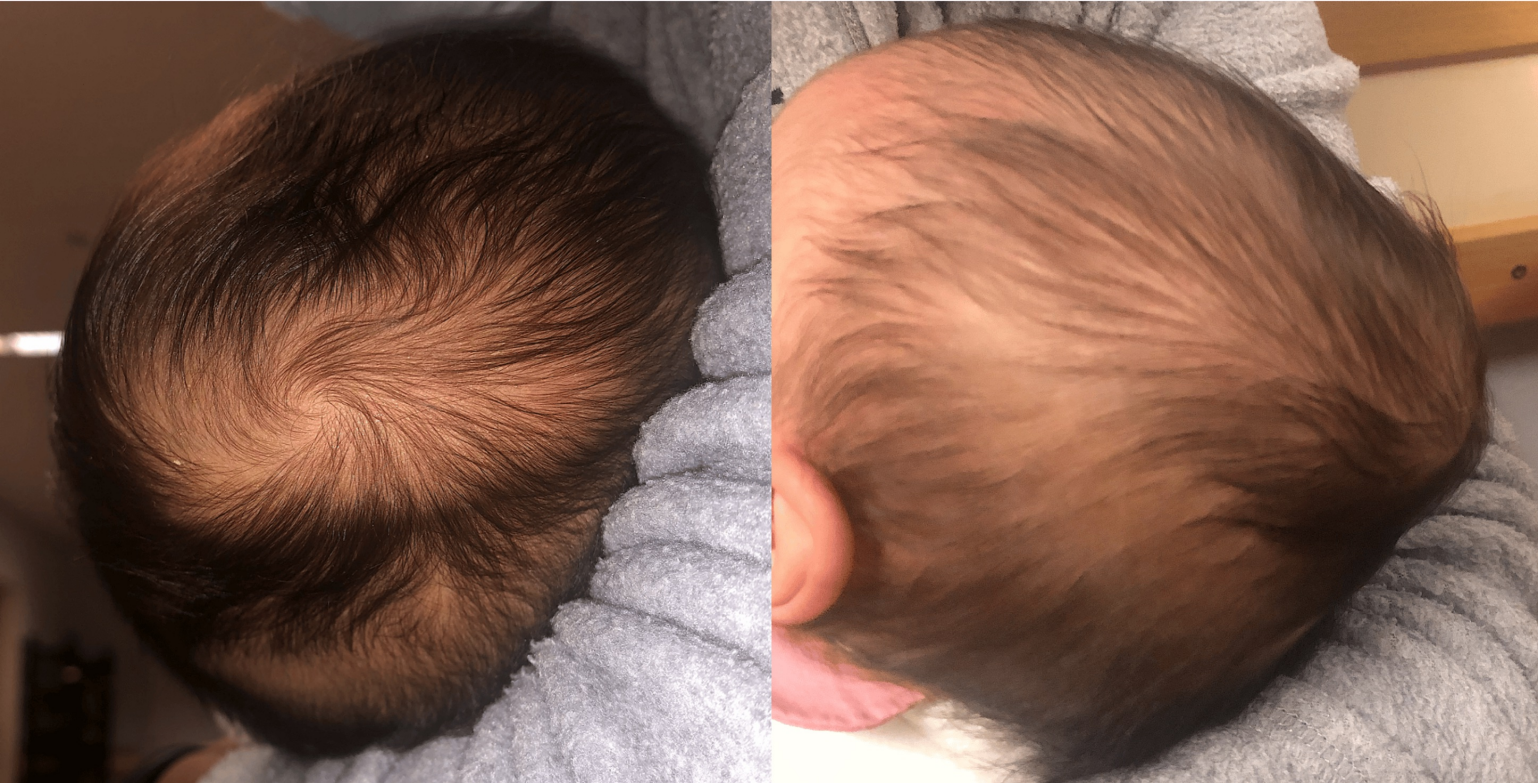
Fluid accumulation in the occipitoparietal region without evidence of overlying scalp erythema or injury. The collection is not limited by the cranial suture lines.

At presentation, she was alert and interactive with normal vital signs. The swelling was oval-shaped, ±4cm in diameter and located predominantly in the right parieto-occipital region. It was soft, non-tender, fluctuant and non-pulsating. It was mobile, not limited by suture lines, and a fluid shift was noted with movement of the head. No inflammatory signs were present (Figure 1). There was no abnormality on palpation of the scalp, and there were no signs of bruising or recent trauma. No other alterations were detected in the physical examination. 

The infant underwent a blood draw (full blood count and clotting profile), skull radiography and cranial ultrasound to ascertain the nature of the swelling and rule out non-accidental injury. The full blood count and clotting profile were normal. Skull radiography revealed a soft tissue swelling, and there was no evidence of fracture. The ultrasound showed an elongated formation of liquid content with a diameter of approximately 3.7 cm, suggesting a simple subgaleal collection. To exclude the hypothesis of child abuse, the ocular fundus was also evaluated, which showed no alterations. Due to the apparent benign nature of clinical findings, the parents were reassured, and the infant was discharged. 

**Figure 2:**
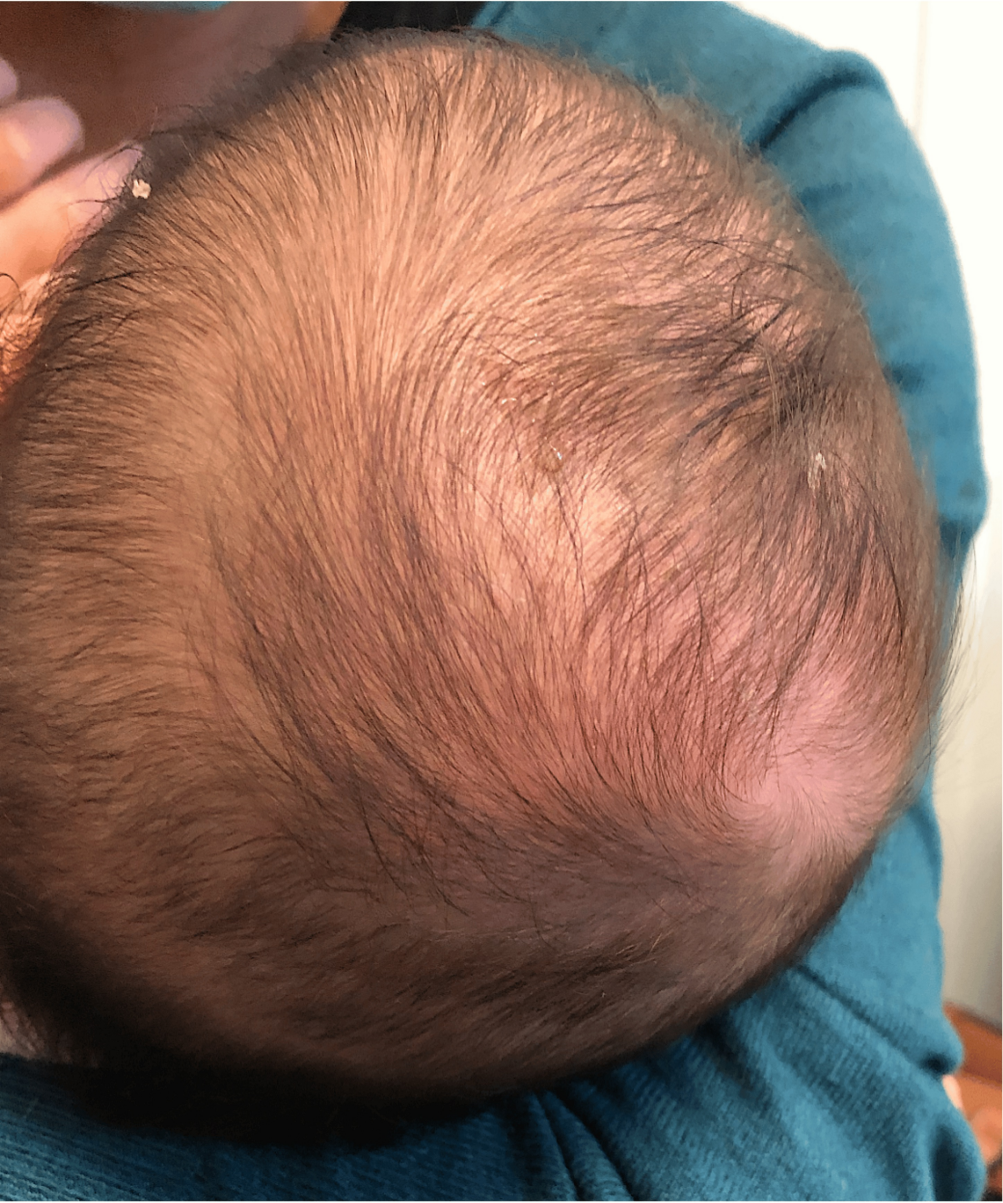
Image at follow-up consultation four weeks after diagnosis with near complete resolution of swelling.

**Figure 3:**
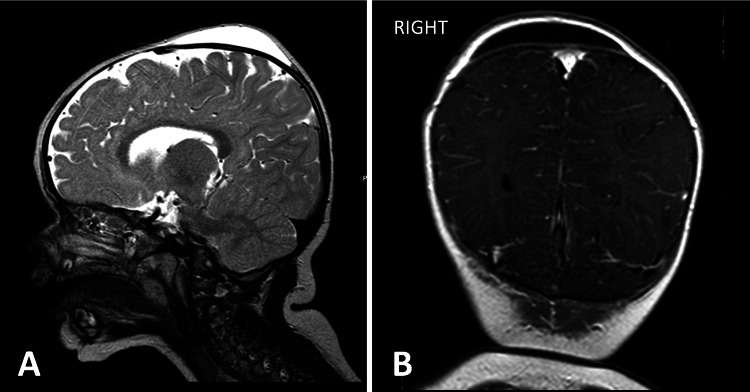
Brain MRI showing presence of biparietoccipital subaponeurotic fluid collection with greater expression on the right, without evidence of skull fracture or intracranial abnormality. (A) Sagittal image. (B) Coronal image.

At a follow-up consultation after four weeks (14 weeks of age), the swelling had almost completely resolved (Figure 2). However, at six months of age, the infant presented with a new scalp swelling with similar characteristics, but this time it was bilateral and located superiorly (biparietoccipital). Cranioencephalic magnetic resonance imaging (MRI) was performed, which showed a bilateral parietal epicranial fluid collection, with greater expression on the right (Figure 3). The fluid collection was identified as subaponeurotic or subgaleal, which was overlying the vertex and parietal region. The brain and extra-axial spaces were unremarkable. The diagnosis of DSFC was hypothesised. After one month, without intervention, a complete and spontaneous resolution of the swelling was observed. To date, no further recurrence has occurred.

## Discussion

DSFC is a rare but increasingly recognised condition typically presenting between two weeks and four months of age [1,5]. The exact mechanism of DSFC formation remains unclear, but several theories have been proposed. One hypothesis suggests CSF leakage through microscopic skull fractures sustained during instrumental or difficult deliveries [7,9]. Another theory involves the disruption of venous or lymphatic drainage in the subaponeurotic space, leading to gradual accumulation of serous fluid [1,13]. Both mechanisms reflect the impact of birth-related trauma on scalp tissue, contributing to delayed fluid collection without acute haemorrhage or infection. Clinically, DSFC appears as a soft, fluctuant, non-tender swelling that crosses suture lines and is not associated with systemic symptoms, inflammation, bruising or signs of trauma [4]. Despite its alarming appearance, DSFC is a benign accumulation of fluid between the galea aponeurosis and the periosteum; it is self-limiting and resolves without intervention in most cases [5].

The infant in our case presented with a unilateral parieto-occipital swelling at eight weeks, which resolved spontaneously within four weeks. The infant’s vacuum-assisted delivery supports the widely reported association between DSFC and instrumental or prolonged labour. Notably, the child later developed a bilateral biparietal recurrence at six months, a feature not frequently reported in the literature. The condition again resolved completely without treatment. Imaging, including ultrasound and MRI, confirmed the fluid collections were confined to the subaponeurotic space, with no underlying structural abnormalities or skull fracture. 

DSFC must be carefully distinguished from other scalp pathologies in infants [4]. Subgaleal haemorrhage is a potentially life-threatening condition that presents immediately after birth, often with bruising and systemic instability [2,10,13]. Cephalohematomas are confined by suture lines, firmer in consistency, and may calcify over time [14-16]. Caput succedaneum also presents at birth, crosses suture lines, and typically resolves within days [2,10,13]. Infectious or inflammatory lesions are characterised by erythema, warmth, and systemic symptoms, which are absent in DSFC [13]. Non-accidental injury should be considered in any unexplained scalp swelling and requires thorough evaluation, including fundoscopy [6,14]. Lastly, congenital cysts or tumours are usually fixed, present from birth, and do not resolve spontaneously [9].

Early and accurate identification of DSFC is essential to avoid unnecessary investigations, referrals, and anxiety for families. In our case, fundoscopy, normal coagulation studies, and imaging findings ruled out serious conditions such as subgaleal haemorrhage or non-accidental injury.

It is essential to offer follow-up consultations to confirm that the fluid collection is gradually decreasing; any persistence, increase in size, or emergence of neurological symptoms should prompt further evaluation [8]. Importantly, MRI was valuable in the setting of recurrence to exclude CSF fistula, a rare but important differential diagnosis when fluid collections persist beyond three months [17]. 

A review of the literature reveals approximately 70 peer-reviewed DSFC cases reported with consistent clinical and radiological profiles. The condition was first described in 2002 by Hopkins et al. in the UK [1] and has since been documented in over a dozen countries (Table 1). Most cases involved healthy infants, with a mean age at diagnosis of eight weeks and a mean resolution time of seven weeks [5,13]. Instrumental deliveries were the most frequently reported risk factor, identified in at least 21 studies. Imaging findings were consistent: ultrasound typically revealed anechoic, mobile collections that crossed suture lines without underlying bone abnormalities, while MRI, reserved for persistent or atypical cases, confirmed the extracranial nature of the collections [7,11,18]. Conservative management was universally effective. Although a few reports described aspiration of the fluid, this approach is now discouraged due to the potential risks of infection and recurrence [1,5,19].

**Table 1:** Comparative summary of peer-reviewed delayed subaponeurotic fluid collection (DSFC) case reports (up to 2025) NR: not referenced; US: ultrasound

Author/Year	Country	No. of Cases/Gender	Age at Diagnosis (Weeks)	Delivery Method/Risk Factors	Imaging	Treatment	Resolution Time (Weeks)
Hopkins et al., 2002 [1]	UK	6/NR	3.5-18 (mean 7)	Instrumental delivery (4)	X-ray, US	Conservative (5); Aspirated twice (1)	2-24 (mean 8.8)
Smith et al., 2016 [2]	Ireland	11/NR	3-12 (mean 7.1)	Instrumental delivery (11)	X-ray, US, CT, MRI	Conservative	1-12 (mean 4)
Valero et al., 2019 [3]	USA	1/girl	5	Cesarean	X-ray, US, MRI	Conservative	1
Mohamed et al., 2025 [4]	USA	1/boy	10	Use of scalp electrodes	US	Conservative	2
Faried et al., 2021 [5]	Indonesia	1/girl	8	Cesarean	US	Aspiration; compression bandage	3.5
Cristina et al., 2023 [6]	Portugal	1/boy	7	Cesarean	US, CT	Conservative	19
Petraglia et al., 2010 [7]	USA	3/NR	5-9 (mean 6.3)	Instrumental delivery (1), use of scalp electrodes (3)	X-ray, CT	Conservative	5-9 (mean 6.7)
Roy et al., 2014 [8]	UK	1/boy	10	Instrumental deliver, use of scalp electrodes	CT, MRI	Aspiration	NR
Schoberer et al., 2008 [9]	Germany	5/2 boys, 1 girl, NR	7-8 (mean 7.8)	Instrumental delivery (4) / NR (1)	US, CT, MRI	Conservative (2); Aspirated (2); Aspirated twice (1)	4-20
Medows et al., 2014 [10]	USA	1/boy	14	Emergency cesarean	CT	Conservative	5
Abusaleem et al., 2024 [11]	Saudi Arabia	1/boy	11	Cesarean	US, CT	Conservative	3
Vaibhav et al., 2010 [12]	UK	4/3 boys, 1 girl	4-14 (mean 7.5)	Instrumental delivery (1)	X-ray, US, MRI	Conservative	3-8 (mean 4.25)
Stephan et al., 2019 [13]	USA	10/5 boys, 5 girls	2-17 (mean 9.7)	Cesarean	X-ray, US, CT	Conservative	NR
Wang et al., 2016 [14]	Canda	9/4 boys, 5 girls	2-11 (mean 5.6)	Instrumental delivery (6)	X-ray, US, CT, MRI	Conservative	2-20 (mean 10.5)
Lee et al., 2018 [15]	USA	1/boy	8	Instrumental delivery	US	Conservative	5
Cullas Ilarslan et al., 2019 [16]	Turkey	5/4 boys, 1 girl	5-14 (mean 9.8)	Instrumental delivery (2)	X-ray, US, CT, MRI	Conservative	2-12 (mean 5.8)
Prasath et al., 2020 [17]	India	2/1 girl, 1 boy	9-11	Cesarean (1)	US	Conservative	6-8
Ribeiro et al., 2024 [18]	Portugal	2/1 boy, 1 girl	7-10	Instrumental delivery (2)	US, CT	Conservative	4
Chalipat et al., 2014 [19]	India	1/girl	4	Emergency Cesarean	MRI	Aspiration	12
Linhares et al., 2021 [20]	Portugal	1/boy	13	Instrumental delivery	X-ray	Conservative	4
Present case	Portugal	1/girl	10	Instrumental delivery	X-ray, US, MRI after recurrence	Conservative	4

As far as we are aware, our case is the first reported to demonstrate recurrence in a different location after initial resolution. This unique presentation adds to the growing body of evidence confirming DSFC as a benign and self-limiting condition, while underscoring the importance of ongoing clinical awareness, appropriate follow-up, and targeted imaging in atypical or recurrent cases. Education of frontline healthcare professionals is essential to ensure timely diagnosis, avoid unnecessary investigations, interventions, or parental anxiety.

## Conclusions

DSFC is a rare, benign cause of scalp swelling in infants that typically presents weeks after birth. It is often associated with instrumental deliveries, such as vacuum or forceps, and usually resolves spontaneously with conservative management. DSFC must be distinguished from more serious conditions like subgaleal haemorrhage or non-accidental injury, to avoid unnecessary investigations or interventions.

This case describes a rare recurrence of DSFC several months after initial resolution, expanding the recognised clinical course of the condition. Bilateral and biparietal involvement, as seen in the recurrence, is less commonly reported in the literature and may challenge clinical expectations. The case reinforces the value of MRI in persistent or atypical presentations and supports a non-invasive approach even in recurrent cases. MRI should be performed to rule out a CSF fistula.

## References

[1] Hopkins RE, Inward C, Chambers T, Grier D (2002). Sub-aponeurotic fluid collections in infancy. Clin Radiol.

[2] Smith A, Kandamany N, Okafor I, Robinson I, Foran A, McNamara R (2016). Delayed infant subaponeurotic (subgaleall) fluid collections: a case series of 11 infants. J Emerg Med.

[3] Valero FD, Castillo C, Prokhorov S, Marino C (2019). An unusual late presentation of swelling over the head. Neoreviews.

[4] Mohamed AA, Sargent EC, Barron J (2025). Spontaneous subaponeurotic fluid collection in an infant: illustrative case. J Neurosurg Case Lessons.

[5] Faried A, Imron A, Aliyannissa A, Indrawati D (2021). Delayed subaponeurotic fluid collection on an infant's head: underreported case and review of the literature. Surg Neurol Int.

[6] Cristina C, Duarte M, Matos M, Machado R (2023). Delayed subaponeurotic fluid collection: an unusual cause of scalp swelling. Nascer Crescer.

[7] Petraglia AL, Moravan MJ, Marky AH, Silberstein HJ (2010). Delayed sub-aponeurotic fluid collections in infancy: three cases and a review of the literature. Surg Neurol Int.

[8] Roy HA, Magdum S (2014). Sub-aponeurotic fluid collection in a neonate associated with fetal scalp electrode monitoring: a brief communication. Eur J Obstet Gynecol Reprod Biol.

[9] Schoberer A, Yagmur E, Boltshauser E, Korinth M, Niggemann P, Häusler M (2008). Sub-aponeurotic fluid collections: a delayed-onset self-limiting cerebrospinal fluid fistula in young infants. Eur J Paediatr Neurol.

[10] Medows M, Mohammad Nijres B (2014). Delayed subaponeurotic (subgaleal) fluid collection. BMJ Case Rep.

[11] Abusaleem MY, Hamouda WO, Abdelshafy MK, Farag AA, Serhan AI (2024). Delayed subaponeurotic fluid collection in a neonate: a case report. Cureus.

[12] Vaibhav A, Smith R, Millman G, Cooper J, Dwyer J (2010). Subaponeurotic or subgaleal fluid collections in infancy: an unusual but distinct cause of scalp swelling in infancy. BMJ Case Rep.

[13] Stephan AM, Feldman KW, Otjen JP, Metz JB (2021). Delayed subaponeurotic fluid collections: a benign cause of scalp swelling in infancy. Pediatr Emerg Care.

[14] Wang S, Drake J, Kulkarni AV (2016). Management and outcome of spontaneous subaponeurotic fluid collections in infants: the Hospital for Sick Children experience and review of the literature. J Neurosurg Pediatr.

[15] Lee JJ, Wenger TL (2018). Delayed subaponeurotic fluid collections of infancy. J Pediatr.

[16] Cullas Ilarslan NE, Gunay F, Kaynak SS, Ucan B, Fitoz OS, Ince E (2019). A rare cause of scalp swelling in infancy: delayed subaponeurotic fluid collections in five cases. Childs Nerv Syst.

[17] Prasath TSA, Gutta MC, Murali A, Ramanan PV (2020). Subaponeurotic fluid collection - an unusual cause of scalp swelling in infancy. Karnataka Paediatr J.

[18] Ribeiro IM, Sousa D, Rocha R, Carvalho C, Godinho C (2024). A rare cause of scalp swelling in infants: two case reports. Cureus.

[19] Chalipat S, Karambelkar G, Dhobale V, Agarkhedkar S, Jadhav R (2014). Sub-aponeurotic fluid collection: a rare cause of scalp swelling in infancy. Pediatr Oncall J.

[20] Linhares MI, Penteado R, Soares GB, Januário L (2021). Delayed infant subaponeurotic (subgaleal) fluid collection. Pediatr Neonatol.

